# Hypomania spectrum disorder in adolescence: a 15-year follow-up of non-mood morbidity in adulthood

**DOI:** 10.1186/1471-244X-14-9

**Published:** 2014-01-15

**Authors:** Aivar Päären, Hannes Bohman, Anne-Liis von Knorring, Lars von Knorring, Gunilla Olsson, Ulf Jonsson

**Affiliations:** 1 Department of Neuroscience, Child and Adolescent Psychiatry, Uppsala University, Uppsala, Sweden; 2 Department of Neuroscience, Psychiatry, Uppsala University, Uppsala, Sweden

**Keywords:** Adolescence, Hypomania spectrum, Follow up, Comorbidity

## Abstract

**Background:**

We investigated whether adolescents with hypomania spectrum episodes have an excess risk of mental and physical morbidity in adulthood, as compared with adolescents exclusively reporting major depressive disorder (MDD) and controls without a history of adolescent mood disorders.

**Methods:**

A community sample of adolescents (N = 2 300) in the town of Uppsala, Sweden, was screened for depressive symptoms. Both participants with positive screening and matched controls (in total 631) were diagnostically interviewed. Ninety participants reported hypomania spectrum episodes (40 full-syndromal, 18 with brief episode, and 32 subsyndromal), while another 197 fulfilled the criteria for MDD without a history of a hypomania spectrum episode. A follow up after 15 years included a blinded diagnostic interview, a self-assessment of personality disorders, and national register data on prescription drugs and health services use. The participation rate at the follow-up interview was 71% (64/90) for the hypomania spectrum group, and 65.9% (130/197) for the MDD group. Multiple imputation was used to handle missing data.

**Results:**

The outcomes of the hypomania spectrum group and the MDD group were similar regarding subsequent non-mood Axis I disorders in adulthood (present in 53 vs. 57%). A personality disorder was reported by 29% of the hypomania spectrum group and by 20% of the MDD group, but a statistically significant difference was reached only for obsessive-compulsive personality disorder (24 vs. 14%). In both groups, the risk of Axis I disorders and personality disorders in adulthood correlated with continuation of mood disorder. Prescription drugs and health service use in adulthood was similar in the two groups. Compared with adolescents without mood disorders, both groups had a higher subsequent risk of psychiatric morbidity, used more mental health care, and received more psychotropic drugs.

**Conclusions:**

Although adolescents with hypomania spectrum episodes and adolescents with MDD do not differ substantially in health outcomes, both groups are at increased risk for subsequent mental health problems. Thus, it is important to identify and treat children and adolescents with mood disorders, and carefully follow the continuing course.

## Background

Owing to the severity of bipolar disorder [1], there is great interest in child and adolescent symptoms of hypomania and whether these symptoms will subsequently develop into bipolar disorder. Long-term follow-up studies have shown different results, depending on the sample and methodology. On the one hand, several clinical samples suggest a continuity of the disorder from childhood and adolescence to early adulthood [2-7]. The continuing course in clinical samples might to some extent be explained by the severity of the disorder and high rates of family history of bipolar disorder in this group.

On the other hand, community samples do not suggest a clear continuity. In a 15-year follow-up of the US Oregon study of high school students aged 14–18 years, only 3.4% of those with subsyndromal hypomania symptoms developed bipolar disorder [8]. An earlier follow-up of the US Oregon study reported that 9 out of the 17 (53%) youths with syndromal bipolar disorder (4 bipolar I, 11 bipolar II, and 2 cyclothymic) failed to recover or had at least one recurrence by the age of 24 years [9]. A prospective longitudinal community sample in Munich [10], of adolescents and young adults aged 14–24 years, found that 7.2% of the participants with subthreshold bipolar disorder at baseline converted to bipolar disorder during a 10 years follow-up, compared to 1.7% with pure MDD. Additionally, in a recent 15-year follow up of a Swedish community sample of adolescents with mood disorders, we found that only 6 out of 64 participants (9%) with hypomania spectrum episodes in adolescence reported either a manic or hypomanic episode in adulthood [11]. Thus, the results suggest that hypomania spectrum episodes in adolescence do not necessarily lead to bipolar disorder. In addition, several longitudinal studies of high-risk offspring with a family history of bipolar disorder indicate a relatively small increased risk of developing bipolar disorders in adulthood [12-16].

However, there might be concerns that hypomanic symptoms in adolescence indicate excess risk of other health conditions. Many epidemiological studies show that comorbidity is common in children and adolescents with syndromal and subsyndromal bipolar disorder [3,17-19]. Child and adolescent bipolar disorder is highly comorbid with attention-deficit/hyperactivity disorder (ADHD), anxiety disorders, obsessive-compulsive disorder (OCD), conduct disorder (CD), post-traumatic stress disorder (PTSD) and substance abuse [20-26]. Also high-risk offspring studies have reported elevated rates of a broad range of psychiatric comorbid disorders, although the rates of ADHD and CD have been relatively low [12,16,27,28].

In adults with bipolar spectrum disorder, high rates of comorbidity for anxiety disorders, substance abuse and personality disorders are reported [29-37]. Recent data also suggest increased comorbid substance/alcohol abuse and anxiety in adults with MDD and subsyndromal hypomanic features, compared to individuals with MDD without a history of subsyndromal hypomanic features [10,38].

Further, personality disorders are frequently associated not only with mood disorders in general but also with bipolar disorder [32,33,35,36,39]. Personality disorders are more common in individuals with an early onset of bipolar disorders than in those with a late onset [39,40]. Some studies suggest that comorbid personality disorders reduce the likelihood of recovery from early-onset forms of mood disorders [37,41]. Also, ADHD and eating disorders are common comorbid disorders in adults with bipolar disorder [31,42], although ADHD seems to be more prevalent in children and adolescents than in adults [20,21,31,43].

Thus, there are reasons to think that the broader spectrum of hypomania in adolescence might be a marker for various kinds of non-mood morbidity. In the present study we investigated the broader health outcomes of adolescents with hypomania spectrum symptoms. In addition to measures of psychiatric non-mood morbidity, register data on health care utilization were used as proxy measures of disease and related health problems. To test the specificity of adolescent hypomania spectrum, we compared this disorder with adolescent MDD without a history of hypomania spectrum episodes. Four main research questions were addressed:

1. Do individuals with a history of hypomania spectrum episodes in adolescence have an excess risk of non-mood Axis I disorders or personality disorders as adults, compared with adolescents with MDD without a history of hypomania spectrum episodes and with controls without a history of adolescent mood disorders?

2. Is the pattern of overlap between anxiety disorders, substance abuse and personality disorders in adults with a history of hypomania spectrum episodes in adolescence similar to or different from that of individuals with a history of MDD in adolescence and that of controls without mood disorders in adolescence?

3. Is the risk of adult non-mood Axis I disorders and personality disorders in individuals with hypomania spectrum episodes or MDD in adolescence related to the continuity of the mood disorders?

4. Do individuals with hypomania spectrum episodes in adolescence utilize more health services and prescription drugs as adults than adolescents with MDD without a history of hypomania spectrum episodes and controls without mood disorders in adolescence?

## Methods

### Study design and participants

The present study is based on a community study carried out during 1991–93 in the Swedish university town of Uppsala (pop. approximately 180 000). All first year students in upper secondary school (16–17 years of age), and school dropouts of the same age, were invited to a screening for depression [44]. Out of a total of 2 465 adolescents in the age group, 2 300 (93%) participated in the screening. Both adolescents with positive screening and controls (in total 631) were diagnostically interviewed.

**Figure 1:**
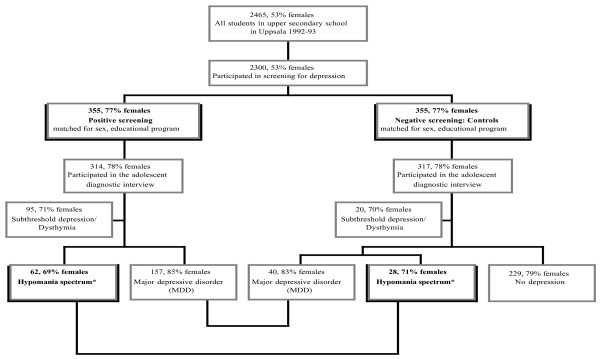
**Chart illustrating the selection of participants and division into groups for the present follow-up study.** *In reference to earlier reports describing this cohort, it should be noted that some subjects previously classified as having MDD or subthreshold depression are here classified as having hypomania spectrum.

In total, 409 (64.6%) of the original participants took part in a follow-up interview after 15 years [45]. Out of the 222 participants who did not take part, 22 (3.5%) had not given their consent to the follow-up study, 39 (6.2%) had emigrated or lived abroad at the time of the follow-up, 3 (0.5%) were not alive, 42 (6.7%) could not be reached, and 116 (18.5%) either refused or could not find the time. The age of the participants ranged between 30 and 33 years (mean = 31.6 years; SD = 0.8) at the time of the follow-up interview. The procedure is outlined in Figure 1.

### Data collection

The screening in adolescence was performed with two self-evaluation instruments for depression: The Beck Depression Inventory-Child (BDI-C) [46] and the Centre for Epidemiological Studies – Depression Scale for Children (CES-DC) [47]. Students with positive screening (BDI ≥ 16 or CES-DC ≥ 30 + BDI ≥ 11, or a previous suicide attempt) were interviewed with the Diagnostic Interview for Children and Adolescents in the revised form according to DSM-III-R for adolescents (DICA-R-A) [48]. For each student with a positive screening, a same sex classmate with negative screening was interviewed in the same manner. In total, 631 adolescents were interviewed and asked for consent to be contacted for a follow-up study.

Fifteen years later, in 2006–2008, a follow-up diagnostic interview was performed with the Mini International Neuropsychiatric Interview Plus (MINI Plus) [49]. At the time of the follow-up interview, the participants also completed the DSM-IV – ICD-10 Personality Questionnaire (DIP-Q) [50]. We also asked the participants for informed consent to link the data from the interviews to data from the Swedish national registers.

### Definition of the groups: Hypomania spectrum, major depressive disorder, and controls

In this study, hypomania was defined as “elevated mood” and/or “grandiosity” and 1–3 additional symptoms, or alternatively, with irritability as the only core symptom and at least four additional symptoms. The reason for this definition was that elevated mood and/or grandiosity can be considered as the “cardinal features” of mania and have been proposed as required symptoms in children and adolescents with hypomania [51]. Thus, only irritable mood was not sufficient as the core symptom unless grandiosity and/or elevated mood were also present [4,51]. This approach was adopted to avoid overlap with dimensional irritability/aggression, which is present across different child and adolescent mental disorders (e.g., ADHD, CD, ODD, MDD, PTSD, and disruptive mood dysregulation disorder).

Based on the base-line evaluation in adolescence, the participants were divided into subgroups as follows:

1. Hypomania spectrum was defined as full-syndromal hypomania (criteria for hypomania were fulfilled for at least 4 days duration), brief-episode hypomania (the symptom criteria for hypomania were fulfilled, but for less than 4 days), or subsyndromal hypomania (1 or 2 main symptoms and 1–2 additional symptoms, with a total of ≥3 symptoms fulfilled; the duration was not defined, but was in most of the cases more than four days).

2. Major Depressive Disorder (MDD) was defined according to DSM-IV criteria.

3. Controls did not fulfill the criteria for MDD or dysthymia, did not screen positive for depression, and did not report a hypomania spectrum episode.

The sample included 90 participants with hypomania spectrum (40 full-syndromal, 18 with brief episode, and 32 subsyndromal), 197 participants with MDD and 229 controls. One adolescent fulfilled the criteria for a manic episode, and was excluded from the analyses.

Participants meeting the criteria for hypomania spectrum both among those with positive and negative screening were categorized as having hypomania spectrum.

Participants with dysthymia or positive screening for depression, but who did not fill the criteria for hypomania spectrum or MDD, were defined as having subthreshold depression and were excluded from the present study (Figure 1). It should be noted that in a previous study of this population, subjects with mania/hypomania were excluded [45]. Therefore, the size of the groups in this study and the previous one are not identical.

### Baseline evaluation

Adolescent mood disorders and other mental disorders were assessed with the DICA-R-A. Previous childhood anxiety disorders, ADHD, ODD, and hypomania were also retrospectively assessed with the DICA-R-A. Additional information on depressive symptoms was available from the BDI-C and CES-DC scores. Among those with hypomania spectrum who had screened positively for depression, 53 met the criteria for a lifetime major depression and 9 had subthreshold depression/dysthymia. Among those with hypomania spectrum who had screened negatively for depression, 15 had a lifetime major depression, 1 had subthreshold depression, and 12 had no depression. To ascertain that the symptoms were not better explained by ADHD, we assessed that the 12 participants in the hypomania group without depression did not meet the criteria for ADHD. The interviews were performed as soon as possible after the screening: 37% within one month, 20% during the second, 14% during the third, 13% during the fourth, 13% during the fifth and sixth months, and 3% later. More than half of the interviews were performed by the same psychiatrist and the remaining by two psychiatry nurses, two psychologists and one student. They were all individually trained by the psychiatrist. In 27 of the interviews, another psychiatrist conducted simultaneous scoring with the interviewer. There were no differences in diagnostic results and only small discrepancies in details. Details about the baseline evaluation have been published previously [44,52].

The proportion of females was 70% in the hypomania spectrum group, compared with 85% in the MDD group and 79% of the controls. The hypomania group and the MDD group differed significantly in this respect (p < 0.05).

The DICA-R-A also included questions about psychosocial stressors in adolescence. There were no significant differences between the hypomania spectrum group and the MDD group regarding economical hardships within the family (8.9 vs. 15.3%), parental unemployment (13.3 vs. 15.2%), and parental divorce (33.3 vs. 31.5%).

The adolescents with mood disorders reported a high comorbidity with axis I disorders: 78% of those with hypomania spectrum episodes reported at least one comorbid diagnosis in adolescence or childhood, compared with 80% of those with MDD and 25% of the controls (Table 1). The adolescents with hypomania spectrum disorder had a significantly higher prevalence of psychotic symptoms (p < 0.05) than the adolescents with MDD, and there was a non-significant trend suggesting that conduct disorder was also more common in the hypomania spectrum group (p = 0.069). For all the other non-mood disorders, the prevalence was similar in the two groups.

**Table 1:** **Mental comorbidity in adolescents with hypomania spectrum episodes or major depressive disorder** (**MDD**) **and in non**-**mood controls**

**Comorbid diagnoses in adolescence (<18 years)**	**Full-syndromal hypomania**^**a **^**n = 40 (%)**	**All hypomania spectrum n = 90 (%)**	**MDD n = 197 (%)**	**Controls n = 229 (%)**	**OR (95% CI)**^**b **^**all hypomania vs. MDD**	**OR (95% CI)**^**b **^**all hypomania vs. controls**
Separation anxiety disorder	20 (50)	32 (36)	77 (39)	26 (11)	0.88 (052–1.48)	4.49 (2.46-8.18)***
Avoidant disorder	7 (18)	14 (16)	23 (12)	4 (2)	1.42 (0.69-2.95)	10.10 (3.21-31.74)***
Overanxious disorder	16 (40)	27 (30)	74 (38)	10 (4)	0.71 (0.41-1.22)	9.42 (4.31-20.58)***
Panic disorder	5 (13)	12 (13)	26 (13)	2 (1)	1.09 (0.52-2.30)	19.96 (4.33-91.94)***
OCD	18 (45)	26 (29)	47 (24)	10 (4)	1.30 (073–2.29)	9.23 (4.20-20.28)***
PTSD	2 (5)	3 (3)	11 (6)	1 (0)	0.57 (0.15-2.11)	7.96 (0.81-78.26)
Psychotic symptoms	4 (10)	9 (10)	6 (3)	-	3.67 (1.25-10.82)*	- ^1^
Anorexia nervosa	-	-	10 (5)	1 (0)	- ^1^	- ^1^
Bulimia nervosa	-	1 (1)	3 (2)	-	0.88 (0.09-8.64)	- ^1^
ADHD	8 (20)	13 (14)	20 (10)	2 (1)	1.24 (0.57-2.70)	17.70 (3.87-80.85)***
CD	12 (30)	29 (32)	39 (20)	10 (4)	1.71 (0.96-3.05)	10.24 (4.72-22.23)***
ODD	4 (10)	8 (9)	20 (10)	2 (1)	0.91 (0.38-2.18)	11.55 (2.39-55.85)**
Drug/sniffing/alcohol abuse	5 (13)	12 (13)	16 (8)	2 (1)	1.56 (0.69-3.52)	18.17 (3.96-83.42)***
**Any comorbidity**	34 (85)	70 (78)	157 (80)	58 (25)	0.89 (0.48-1.65)	10.56 (5.88-18.97)***

* <0.05; ** <0.01; *** < 0.001.

^a^Full-syndromal hypomania is included in all hypomania spectrum, but is also presented separately for transparency.

^b^All p-values adjusted for sex in a logistic regression model.

^1^Adjustment for sex was not possible owing to missing symptoms in comparison group.

MDD: Major Depression; OCD: Obsessive-compulsive disorder; PTSD: Posttraumatic stress disorder; ADHD: Attention-deficit/hyperactivity disorder; CD: Conduct disorder; ODD: Oppositional defiant disorder; OR: Odds ratio; CI: Confidence interval.

Within the hypomania spectrum group, comorbidity did not differ much between males and females, with the exception that boys had a higher prevalence of ADHD (26 vs. 10%; χ^2^ = 4.1; p < 0.05).

### Follow-up evaluation

At age 30–33 years, the participants were diagnostically interviewed with the MINI Plus. Some non-mood disorders were assessed from age 19 years to follow up (panic disorder and agoraphobia, alcohol abuse or dependence, and drug abuse or dependence), while only current status at follow-up was assessed for the other non-mood disorders.

Five interviewers trained in clinical psychology or psychiatry conducted the follow-up interviews. They were blinded to which subgroup the participants belonged. To enhance inter-rater reliability, one interview for each interviewer was video-recorded and rated by the other interviewers. These recordings yielded an overall (for all the included diagnoses in MINI) free-marginal kappa value of 0.93. To further increase reliability, and to ensure the clinical validity of the diagnoses, uncertainties were regularly discussed with senior psychiatrists during sessions of group supervision.

### Attrition to follow-up

The participant rate at follow-up for the total hypomania spectrum group was 71% (64/90): 67.5% (27/40) for full-syndromal hypomania, 66.7% (12/18) for brief-episode hypomania and 78.1% (25/32) for subsyndromal hypomania. Participation rate was 65.9% (130/197) for the MDD group, and 64.6% (148/229) for the control group. The participation rate did not differ significantly between the groups. To assess dissimilarities between those who were lost to follow-up and those who participated in each group, these two groups were compared regarding child and adolescent mental disorders and sex. The groups were similar, with the exception that OCD was significantly more common among those who did not participate in the follow-up (46 vs. 22 %; χ^2^ = 5.31; p = 0.021) (Additional file 1: Table S1).

### The Swedish national registers

The National Board of Health and Welfare keeps the official registers concerning health and sickness in Sweden. The Patient Register contains psychiatric in-patient care on a national level since 1973, all in-patient care since 1987, and outpatient care (primary care excluded) since 2001. The purpose of this register is to follow the development of health in the population, to obtain information on health care use, to improve the abilities of prevention and treatment of disease and to contribute to the progress of health care.

The Prescribed Drug Register contains information on all dispensed prescribed drugs to the entire Swedish population from 1999 and onwards. The personal identity number has been available since July 2005. The quality is regarded as very good, and only 0.3% of the records lack information on the personal identity number. All drugs are classified according to the Anatomical Therapeutic Chemical (ATC) classification system. Measurement units of use are prescriptions, Defined Daily Doses (DDDs) and expenditures. Updates are carried out monthly.

After informed consent, we obtained data on registered inpatient care (including delivery) during the years 1992 through 2009 and outpatient care during the years 2001 through 2009 with the diagnoses according to the International Classification of Diseases, 10th edition (ICD-10). The diagnoses were grouped according to the chapters in ICD-10, except for Chapter V, mental and behavioral disorders, where each block was grouped and analyzed separately.

Following the same procedure, data on prescription drugs during the years 2005 through 2009 were obtained and categorized according to the ATC. The drugs were grouped according to the ATC main groups, with the exception of the nervous system (N01-N07) in which each pharmacological/therapeutic subgroup was grouped and analyzed separately.

Data was obtained for 64.4% (58/90) of the hypomania spectrum group, 64.5% (127/197) of the MDD group, and 64.2% (147/229) of the controls.

### Statistical analyses

The baseline characteristics and outcomes in the three groups (hypomania spectrum, MDD and controls) were compared by univariate logistic regression analyses. As the hypomania spectrum group had a significantly higher proportion of males than the MDD group at baseline, all analyses were also done with logistic regression adjusted for sex. Only the adjusted odds ratios (ORs) with 95% confidence intervals (CIs) are presented.

Owing to skew distributions, differences between means were tested with of the Mann Whitney U test. The subgroups of hypomania spectrum (full-syndromal, brief episode, and subsyndromal) were deemed to be too small to be analyzed separately. However, all background characteristics and outcomes are presented separately for the participants with full-syndromal hypomania, to increase transparency.

To address the problem with loss of information and possible bias because of attrition, a multiple imputation approach was adopted for the logistic regression analyses.

Missing values on all outcome variables were imputed with a model consisting of group (hypomania spectrum, MDD, and controls) and all the variables presented in Table 1. Following the recommendations of Graham and colleagues [53], twenty data sets were imputed; the pooled estimates of these were computed. In addition, complete-cases analyses of all models were conducted.

P-values of less than 0.05 were reported as significant. All statistical analyses were performed with the SPSS 21.0.

### Ethics

The study was approved by the local ethical vetting board of Uppsala University, Sweden.

## Results

### Non-mood Axis I disorders at follow-up

A non-mood disorder on Axis I was reported at follow-up by 34 (53%) of the hypomania spectrum group, compared with 74 (57%) of the MDD group, and 41 (28%) of the controls (Table 2).

**Table 2:** Adult non-mood Axis I disorders in a 15-year follow-up of adolescents with hypomania spectrum disorder or major depressive disorder (MDD) and of non-mood controls

**Non-mood Axis I disorders in adulthood (MINI)**	**Full-syndromal hypomania**^**a **^**n = 27 (%)**	**All hypomania spectrum n = 64 (%)**	**MDD n = 130 (%)**	**Controls n = 148 (%)**	**Followed cases**		**Multiple imputation data**	
					**OR (95% CI)**^**d **^**all hypomania vs. MDD**	**OR (95% CI)**^**d **^**all hypomania vs. controls**	**OR (95% CI)**^**d **^**all hypomania vs. MDD**	**OR (95% CI)**^**d **^**all hypomania vs. controls**
GAD^b^	6 (22)	12 (19)	19 (15)	7 (5)	1.51 (0.67-3.39)	5.15 (1.90-13.97)***	1.36 (0.64-2.90)	5.72 (2.25-14.58)***
Panic disorder^c^	6 (22)	14 (22)	23 (18)	11 (7)	1.55 (0.72-3.33)	3.82 (1.61-9.08)**	1.77 (0.87-3.57)	5.01 (2.22-11.29)***
Social anxiety^c^	5 (19)	11 (17)	27 (21)	10 (7)	0.75 (0.34-1.64)	2.79 (1.11-6.99)*	0.61 (0.29-1.27)	3.36 (1.37-8.27)**
Agoraphobia^c^	6 (22)	14 (22)	28 (22)	12 (8)	1.14 (0.55-2.39)	3.48 (1.49-8.15)**	1.03 (0.54-1.98)	4.23 (1.94-9.21)***
OCD^b^	-	4 (6)	6 (5)	2 (1)	1.67 (0.45-6.21)	5.62 (0.99-31.81)	1.42 (0.38-5.32)	4.83 (0.87-27.80)
PTSD^b^	-	4 (6)	1 (1)	-	8.97 (0.97-83.25)	- ^1^	9.20 (1.06-79.93)*	- ^1^
Somatization disorder^b^	-	1 (2)	3 (2)	-	0.59 (0.07-5.47)	- ^1^	0.64 (0.07-5.83)	- ^1^
Dysmorphophobia^b^	1 (4)	3 (5)	5 (4)	1 (1)	1.48 (0.34-6.46)	8.30 (0.84-81.98)	1.12 (0.26-4.95)	8.66 (0.89-84.49)
Bulimia nervosa^b^	1 (4)	3 (5)	2 (2)	-	3.80 (0.61-23.55)	- ^1^	8.66 (1.66-48.09)*	- ^1^
Psychotic symptoms^b^	-	-	2 (2)	2 (1)	- ^1^	- ^1^	- ^1^	- ^1^
ADHD^b^	-	2 (3)	1 (1)	1 (1)	3.85 (0.33-44.89)	4.55 (0.40-51.84)	2.93 (0.24-36.02)	4.82 (0.44-52.41)
Alcohol abuse^c^	4 (15)	10 (16)	17 (13)	11 (7)	1.29 (0.55-3.05)	2.19 (0.87-5.49)	1.39 (0.67-2.87)	3.52 (1.53-8.10)**
Drug abuse^c^	-	4 (6)	8 (6)	3 (2)	2.00 (0.27-15.00)	5.41 (0.48-61.15)	3.75 (0.72-19.53)	14.79 (1.70-128.94)*
**Any non-mood morbidity**	15 (56)	34 (53)	74 (57)	41 (28)	0.86 (0.46-1.58)	2.98 (1.62-5.50)***	0.85 (0.48-1.51)	4.04 (2.29-7.16)***

* <0.05; ** <0.01; *** < 0.001.

^a^Full-syndromal hypomania in the follow up is included in all hypomania spectrum, but is also presented separately for transparency.

^b^Current at follow-up.

^c^Any time from age 19 to follow-up.

^d^All p-values adjusted for sex in a logistic regression model.

^1^Missing cases in the comparison group; could therefore not be analyzed.

MINI: MINI International Neuropsychiatric Interview Plus; MDD: Major depressive disorder; GAD: Generalized anxiety disorder; OCD: Obsessive-compulsive disorder; PTSD: Posttraumatic stress disorder; ADHD: Attention-deficit/hyperactivity disorder; OR: Odds ratio; CI: Confidence interval.

The most prevalent disorders were anxiety disorders. There were no significant differences between the hypomania spectrum group and the MDD group regarding any of the investigated Axis I disorders in the complete cases analyses. In the analyses with multiple imputations, no significant differences were found except for PTSD and Bulimia nervosa, although the prevalence of these disorders was low (Table 2).

### Personality disorders at follow-up

A personality disorder in adulthood was reported by 18 (29%) of the participants with hypomania spectrum in adolescence, compared with 26 (20%) of those with adolescent MDD, and 12 (8%) of the controls (Table 3).

**Table 3:** Adult personality disorders in a 15-year follow-up of adolescents with hypomania spectrum disorder or major depressive disorder (MDD) and of non-mood controls

**Personality disorders (DIP-Q)**	**Full-syndromal hypomania**^**a **^**n = 26 (%)**	**All hypomania spectrum n = 62 (%)**	**MDD n = 133 (%)**	**Control n = 149 (%)**	**Followed cases**		**Multiple imputation data**	
					**OR (95% CI)**^**b **^**all hypomania vs. MDD**	**OR (95% CI)**^**b **^**all hypomania vs. controls**	**OR (95% CI)**^**b **^**all hypomania vs. MDD**	**OR (95% CI)**^**b **^**all hypomania vs. controls**
**Cluster A** (odd or eccentric)								
**Any cluster A disorder**	7 (27)	13 (21)	16 (12)	5 (3)	1.98 (0.88-4.46)	7.54 (2.55-22.26)***	1.59 (0.69-3.69)	6.39 (2.10-19.49)**
Paranoid personality	5 (19)	10 (16)	12 (9)	4 (3)	1.96 (0.79-4.86)	6.73 (2.02-22.50)**	1.40 (0.64-3.05)	8.02 (2.39-26.88)***
Schizoid personality	-	1 (2)	3 (2)	1 (1)	0.83 (0.08-8.19)	2.64 (0.16-43.18)	0.84 (0.09-8.06)	2.96 (0.18-48.46)
Schizotypal personality	3 (12)	8 (13)	9 (7)	4 (3)	2.07 (0.75-5.73)	5.34 (1.54-18.53)**	2.07 (0.76-5.60)	5.50 (1.59-19.04)**
**Cluster B** (dramatic, emotional, or erratic)								
**Any cluster B disorder**	2 (8)	6 (10)	18 (14)	4 (3)	0.68 (0.26-1.84)	4.12 (1.11-15.26)*	0.69 (0.25-1.88)	4.03 (1.12-14.48)*
Antisocial personality	1 (4)	2 (3)	2 (2)	-	2.09 (0.28-15.64)	- ^1^	1.94 (0.26-14.22)	- ^1^
Borderline personality	2 (8)	6 (10)	14 (11)	4 (3)	0.98 (0.35-2.71)	4.12 (1.11-15.26)*	0.89 (0.36-2.21)	4.98 (1.40-17.68)*
Histrionic personality	1 (4)	3 (5)	3 (2)	-	1.97 (0.38-10.36)	- ^1^	1.67 (0.33-8.56)	- ^1^
Narcissistic personality	-	2 (3)	5 (4)	-	1.07 (0.86-1.34)	- ^1^	0.86 (0.17-4.43)	- ^1^
**Cluster C** (anxious or fearful)								
**Any cluster C disorder**	9 (35)	18 (29)	23 (17)	12 (8)	1.85 (0.90-3.80)	4.61 (2.06-10.33)***	1.69 (0.84-3.40)	4.25 (1.90-9.50)***
Avoidant personality	5 (19)	11 (18)	17 (13)	6 (4)	1.43 (0.62-3.30)	5.14 (1.80-14.66)**	1.15 (0.52-2.54)	4.90 (1.69-14.18)**
Dependent personality	2 (8)	2 (3)	6 (5)	1 (1)	0.83 (0.16-4.25)	5.41 (0.48-61.15)	0.48 (0.10-2.24)	5.54 (0.49-62.76)
Obsessive-compulsive personality	7 (27)	15 (24)	19 (14)	11 (7)	1.83 (0.85-3.95)	3.90 (1.67-9.12)**	2.29 (1.13-4.64)*	5.42 (2.44-12.05)***
**Any personality disorder**	9 (35)	18 (29)	26 (20)	12 (8)	1.61 (0.80-3.26)	4.61 (2.06-10.33)***	1.74 (0.92-3.31)	6.63 (2.93-15.00)***

* <0.05; ** <0.01; *** < 0.001.

^a^Full-syndromal hypomania in the follow up is included in all hypomania spectrum, but is also presented separately for transparency.

^b^All p-values adjusted for sex in a logistic regression model.

^1^Missing cases in the comparison group; could therefore not be analyzed.

DIP-Q: DSM-IV – ICD-10 Personality Questionnaire; MDD: Major depressive disorder; OR: Odds ratio; CI: Confidence interval.

A cluster A disorder in adulthood was reported by 13 (21%) of the participants with hypomania spectrum in adolescence, compared with 16 (12%) of those with adolescent MDD, and 5 (3%) of the controls. A cluster B disorder in adulthood was reported by 6 (10%) of the participants with hypomania spectrum in adolescence, compared with 18 (14%) of those with adolescent MDD, and 4 (3%) of the controls. A cluster C disorder in adulthood was reported by 18 (29%) of the participants with hypomania spectrum in adolescence, compared with 23 (17%) of those with adolescent MDD, and 12 (8%) of the controls.

The most prevalent personality disorders in the hypomania spectrum group were obsessive compulsive (24%), avoidant (18%), and paranoid personality disorder (16%). There were few cases of borderline (10%), antisocial (3%), and schizotypal personality disorder (2%). No significant differences were found between the hypomania spectrum group and the MDD group in the complete-cases analyses. In the analyses with multiple imputation, no significant differences were observed between the hypomania spectrum and MDD groups with the exception of obsessive-compulsive personality disorder which was significantly more prevalent in the hypomania spectrum group (Table 3).

### Overlap of anxiety disorders, substance abuse and personality disorders at follow-up

**Figure 2:**
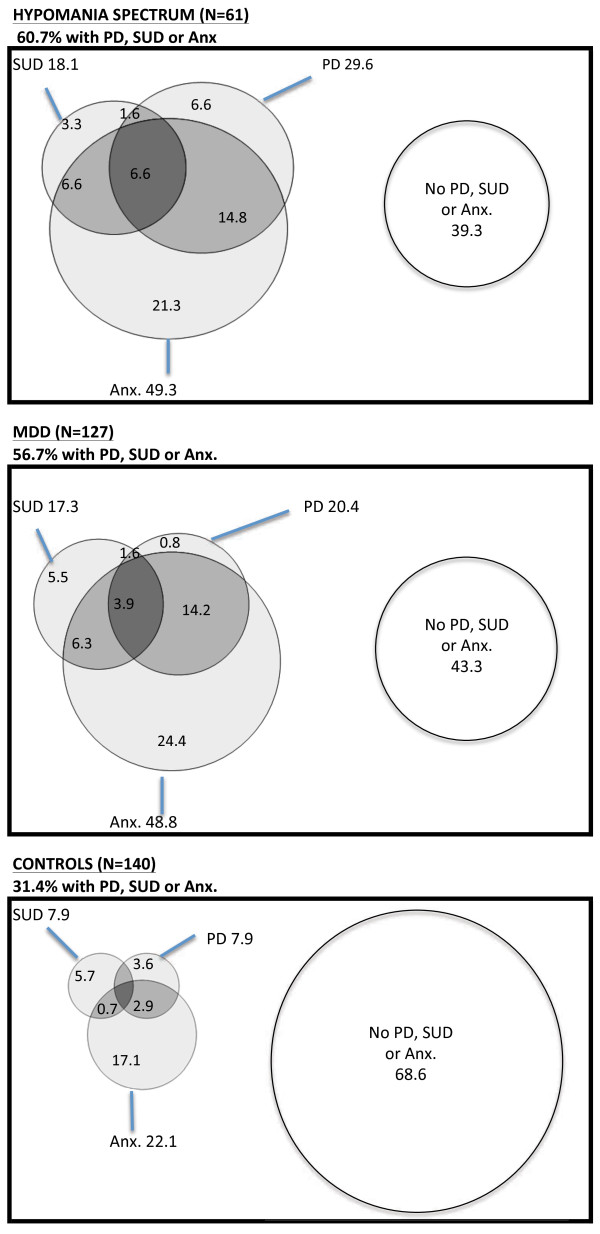
**Venn diagrams illustrating the overlap of adult anxiety disorders (Anx.), substance use disorders (SUD) or personality disorder (PD) in former adolescents with hypomania spectrum or major depressive disorder (MDD) and in controls.** All values shown as %.

Venn diagrams were used to illustrate how anxiety disorders, substance abuse, and personality disorders overlapped in the three groups at follow up (Figure 2). The pattern of overlap was similar for the hypomania spectrum group and the MDD group. In the control group, these disorders were less common and the overlap of the disorders was less extensive.

### Association between continued mood disorders and adult non-mood disorders

Continued mood disorder in adulthood (MDD, bipolar disorder, or dysthymia) was reported by 60.9% of the hypomania spectrum group and by 70.0% of the MDD group. Within the hypomania spectrum group, an adult non-mood disorder on Axis I was reported by 74% of those with continued mood disorder and 24% of those with no continued mood disorder (OR 9.18; CI 95% 2.86 to 29.46). An adult personality disorder was reported by 42% of those with continued mood disorder, and by 9% of those without continued mood disorder (OR 7.64; CI 95% 1.56 to 37.33).

Within the MDD group, an adult non-mood disorder on Axis I was reported by 73% of those with continued mood disorder and 31% of those with no continued mood disorder (OR 5.94; CI 95% 2.61 to 13.50). An adult personality disorder was reported by 26% of those with continued mood disorder, and by 8% of those without continued mood disorder (OR 4.07; CI 95% 1.14 to 14.49).

### Health service use and prescription drugs in adulthood

The mean number of outpatient visits of the hypomania spectrum group during follow-up did not differ significantly from the MDD group (OR 11.7; 95% CI 8.6 to 14.8 vs. OR 12.3; 95% CI 9.7 to 15; n.s.), but was significantly higher than in the control group (OR 6.8; 95% CI 5.2 to 8.4; p < 0.01). The mean number of inpatient visits in adulthood was similar in the hypomania spectrum group (OR 2.3; 95% CI 1.7 to 3.0), the MDD group (OR 2.8; 95% CI 2.1 to 3.6) and the control group (OR 1.8; 95% CI 1.4 to 2.2). The proportion that had been treated for a mental disorder did not differ between the hypomania spectrum group and the MDD group (24 vs. 21%; n.s.), but was lower in the control group (24 vs. 7%; p < 0.001). For the specific blocks within ICD-10 Chapter V, mental and behavioral disorders, only the hypomania group differed from the control group concerning the block of neurotic, stress-related and somatoform disorders (Table 4).

**Table 4:** **Adult in**- **or outpatient care** (**ICD**-**10 codes**) **and prescription drugs** (**ATC groups**) **in a 15**-**year follow**-**up of adolescents with hypomania spectrum disorder or major depressive disorder** (**MDD**) **and of non**-**mood controls**

**Health service use and prescription drugs**^**a**^	**Full-syndromal hypomania**^**b **^**n = 25 (%)**	**All hypo-mania spectrum n = 58 (%)**	**MDD n = 127 (%)**	**Controls n = 147 (%)**	**Followed cases**		**Multiple imputation data**	
					**OR (95% CI)**^**c **^**all hypomania vs. MDD**	**OR (95% CI)**^**c **^**all hypomania vs. controls**	**OR (95% CI)**^**c **^**all hypomania vs. MDD**	**OR (95% CI)**^**c **^**all hypomania vs. controls**
***In- or outpatient care due to mental disorders***								
**F10-19** Substance related dis.	1 (4)	2 (3)	3 (2)	1 (1)	1.18 (0.18-7.69)	4.35 (0.38-50.31)	1.12 (0.18-6.85)	4.86 (0.43-55.16)
**F20-29** Schizophrenia and other psychotic dis.	-	1 (2)	1 (1)	-	1.64 (0.09-29.21)	- ^1^	1.36 (0.08-22.43)	- ^1^
**F30-39** Mood dis.	1 (4)	6 (10)	11 (9)	8 (5)	1.41 (0.49-4.07)	2.31 (0.75-7.11)	1.64 (0.67-4.02)	3.97 (1.42-11.09)**
**F40-48** Anxiety and somatoform dis.	1 (4)	9 (16)	14 (11)	5 (3)	1.89 (0.75-4.77)	6.22 (1.95-19.83)**	2.10 (0.91-4.84)	8.20 (2.82-23.87)***
**F50-59** Eating dis., sleep dis., sexual dysfunct.	-	2 (3)	3 (2)	2 (1)	1.45 (0.23-9.27)	2.93 (0.40-21.45)	2.70 (0.58-12.67)	6.24 (1.02-38.02)*
**F60-69** Personality dis.	-	-	4 (3)	2 (1)	- ^1^	- ^1^	- ^1^	- ^1^
***In- or outpatient care due to physical diseases***								
**K00-K93** Diseases of the digestive system	7 (28)	15 (26**)**	23 (18)	18 (12)	1.64 (0.77-3.48)	2.44 (1.13-5.28)*	1.75 (0.87-3.52)	3.38 (1.69-6.79)***
**S00-S98** Injury, poisoning and certain other consequences of external causes	6 (24)	22 (38)	41 (32)	31 (21)	1.17 (0.60-2.27)	2.14 (1.08-4.26)*	1.65 (0.93-2.90)	3.78 (2.09-6.86)***
***Prescription drugs***								
**L.** Antineoplastic and immunomodulating agents	1 (4)	4 (7)	1 (1)	1 (1)	9.72 (1.04-90.78)*	10.17 (1.10-93.78)*	9.25 (0.98-87.32)	11.17 (1.13-110.00)*
**N. Nervous system**	9 (36)	29 (50)	74 (58)	59 (40)	1.01 (0.79-1.30)	1.59 (1.19-2.13)**	1.11 (0.62-1.97)	3.23 (1.87-5.57)***
N03 Anticonvulsants	-	-	6 (5)	1 (1)	- ^1^	- ^1^	- ^1^	- ^1^
N05a Antipsychotics	1 (4)	4 (7)	2 (2)	-	4.90 (0.85-28.14)	- ^1^	7.82 (1.53-39.92)*	- ^1^
N05b Anxiolytics	4 (16)	11 (19)	15 (12)	6 (4)	2.02 (0.85-4.82)	5.74 (2.00-16.50)***	1.80 (0.83-3.87)	7.14 (2.88-17.73)***
N05c Hypnotics and sedatives	-	9 (16)	21 (17)	13 (9)	0.82 (0.34-1.96)	1.89 (0.76-4.72)	0.91 (0.42-1.94)	2.57 (1.11-5.93)*
N06a Antidepressants	6 (24)	18 (31)	35 (28)	19 (13)	1.31 (0.65-2.62)	3.06 (1.46-6.42)**	1.33 (0.72-2.46)	4.74 (2.43-9.22)***
N06b Psychostimulants	-	-	3 (2)	-	- ^1^	- ^1^	- ^1^	- ^1^
N07 Drugs for addictive disorders	-	2 (3)	6 (5)	-	0.88 (0.17-4.52)	- ^1^	0.70 (0.14-3.35)	- ^1^

* <0.05; ** <0.01; *** < 0.001.

^a^Only diagnostic groups with significant group differences or of specific interest are shown.

^b^Full-syndromal hypomania in the follow up is included in all hypomania spectrum, but is also presented separately for transparency.

^c^All p-values adjusted for sex in a logistic regression model.

^1^Missing cases in the comparison group; could therefore not be analyzed.

ICD-10: International Statistical Classification of Diseases and Related Health Problems, Tenth Revision; ATC groups: Anatomic Therapeutic Chemical classification system; MDD: Major depressive disorder; OR: Odds ratio; CI: Confidence interval.

The proportions that had been treated for a somatic disorder were 83% in the hypomania spectrum group, 86% in the MDD group, and 80% in the control group. The hypomania spectrum group did not differ from the MDD and controls, with two exceptions: Diseases of the digestive system and injury, poisoning and certain other consequences of external causes (including attempted suicide) were less common in the control group (Table 4).

In the complete-cases analyses, there were no significant differences between the hypomania spectrum and MDD groups regarding the proportions that had received prescription drugs, with the exception that a larger proportion of the hypomania spectrum group received prescriptions for antineoplastic and immunomodulating agents. Both groups were more likely than the controls to be prescribed antidepressants, anxiolytics, antipsychotics and drugs for addictive disorders. In the analyses with multiple imputation, similar estimates were obtained, with the exception that antipsychotics were prescribed to a significantly larger proportion of the hypomania spectrum group compared with the MDD group (Table 4).

## Discussion

This 15 year follow-up of a community sample presents unique results of longitudinal health outcomes of adolescents with hypomania spectrum episodes. In a recent publication, we reported that adolescents with hypomania spectrum disorders and adolescents with MDD had a similar risk of continued mood disorders as adults [11]. The present study uses the same community sample to investigate if hypomania spectrum in adolescence is a marker of future excess risk for other mental disorders, high consumption of prescription drugs, and high health care use. The general pattern of anxiety disorders, personality disorders, and substance use disorders in adulthood was similar in the two groups, but clearly differed from that of the control group. Thus, the results suggest that adolescent hypomania spectrum and MDD do not appear to constitute two distinct subgroups in this respect. Rather, they seem to overlap on an affective spectrum scale.

Our results might be surprising given previous research on the extensive comorbidity with bipolar disorder in both adolescents and adults. Mental disorders such as anxiety disorders, ADHD, and CD might either precede or coexist with bipolar disorder [2,3,54-57]. In addition, bipolar spectrum disorders might also be difficult to differentiate from ADHD in children and adolescents.

Despite the fact that both the hypomania spectrum and MDD groups had high prevalences of adolescent externalizing disorders (CD/ODD/ADHD), few participants met the criteria for ADHD as adults. This seems to be in line with previous follow-up studies of hyperactive children with bipolar disorder, which have not documented a significant increased risk of comorbid diagnoses of ADHD in adults [57-59]. Instead, externalizing disorders with hypomania spectrum might predict subsequent cluster B personality disorders and other affective disorders, but not necessarily bipolar disorders [32,37,60,61]. Although hypomania spectrum and externalizing disorders coexist in adults [31,43,59,62], this seems to be considerably less common than in children and adolescents [20,21].

Alcohol and substance related problems in the hypomania spectrum and MDD groups were low both in adolescence and adulthood, despite the co-occurrence of mood and substance use disorders in previous community samples [63-65]. The surprisingly low rate of substance use disorders could be the result of a low response-rate among individuals with these disorders or of low rates of substance use in Sweden in general [66].

The hypomania spectrum group and the MDD group had a similarly high rate of anxiety disorders, both in adolescence and in adulthood. It has been reported that MDD is comorbid with anxiety disorders in general [67,68], while comorbid panic disorder and PTSD is especially prevalent with bipolar II disorder [36,69-72]. However, neither in adolescence nor adulthood did we find a higher prevalence of panic disorder in the hypomania spectrum group. PTSD was only reported by a small proportion in both adolescence and adulthood, precluding any conclusions about differences between the groups. Similarly, the prevalence of bulimia nervosa was low considering the higher rates of comorbidity with bipolar II and eating disorders in some previous studies [42,71-73].

Both the hypomania spectrum group and the MDD group also had a high risk of personality disorders as adults. Previous research has reported overlap between bipolar spectrum and cluster A and B disorders such as borderline, antisocial and schizotypal [32,33,39]. Cluster C disorders have been reported to be more typical of MDD [74]. In our sample, personality disorders within cluster C (obsessive-compulsive and avoidant) were the most prevalent in both the hypomania spectrum group and MDD group, while only a few cases with borderline, antisocial and schizotypal personality disorders were found. The hypomania spectrum group, and in particular the group with full-syndromal hypomania in adolescence, seemed to have a somewhat higher rate of paranoid and obsessive-compulsive personality disorder than the MDD group. However, only for obsessive-compulsive personality disorder was a statistically significant difference observed.

Finally, health service use and prescription drugs in adults were similar in the hypomania spectrum and MDD groups, which further emphasizes that the severity of the conditions in these groups are similar. However, the increased use of mental health services and psychotropic drugs in both groups underscores the continuity of mental and behavioral disorders in adolescents with mood disorders. This confirms the results from other prospective studies showing that child and adolescent onset mood disorders are associated with higher rates of treatment seeking and use of psychotropic drugs [75,76].

Our results show that the continued course of adolescent mood disorders is strongly associated with non-mood morbidity in adulthood. From the clinical perspective, it is therefore important to identify and treat children and adolescents with mood disorders and to monitor their continuing course. About two-thirds of adults with mood disorders report an onset during childhood and adolescence [54,77], and children and adolescents are often diagnosed many years after the onset of the illness. This might lead to a longer duration of untreated illness, increased risks of chronicity and social impairment, and higher health care costs.

In summary, less severe symptoms of hypomania in adolescence do not seem to constitute a specific marker of continued health problems. These results, in combination with our previous report on the continuity of mood disorders in this group, cast doubt on the diagnostic usefulness of a broader spectrum of hypomania in adolescence. In contrast, short-duration hypomania and subsyndromal bipolar disorder in adults seems to be associated with greater comorbidity than MDD [38,78]. It would be of great interest to further investigate this potential distinction between adolescents and adults. For instance, developmental aspects might be important to consider with respect to the potential diagnosis of Depressive Episode with Short-Duration Hypomania that recently was included in the section for conditions for further study in DSM-V [78].

It is possible that other clinical characteristics are better predictors of the continued course of psychopathology in adolescents with mood disorders. We have previously reported that long duration of depression [45] and somatic symptoms [79] in adolescence predict continued mood disorder and other forms of psychopathology in adulthood, suggesting that such factors might be more important than hypomanic symptoms per se in this age group. Much has been published on this topic in the past few years [12,13,27,28], although specific predictors have been difficult to find. Therefore, more longitudinal studies are needed to further explore predictors of poor outcome of adolescent mood disorders.

Some limitations should be considered. Of the source population of 2465 adolescents, 93.3% participated in the original screening [44]. However, out of the 183 high-school dropouts who were invited, only 86 (53%) participated in the screening.

A total of 29% of the subjects in the hypomania spectrum group were lost to follow-up. Although the participation rate at follow-up did not differ between the groups, the subjects lost to follow-up could for instance represent a group with more severe disorders. However, analyses of adolescent characteristics suggest that those lost to follow-up and those who participated had similar psychopathology in adolescence [11]. We also had access to register data on inpatient and outpatient care and prescription drugs use for 97% of the original participants [80,81]. These data were not available separately for the hypomania spectrum group, but showed that only a few received inpatient or outpatient care for mental disorders. Thus, it is unlikely that we have missed a large number of individuals with severe conditions.

Except for inpatient care, the register data on specialized outpatient care and the register data on prescription drugs do not cover the whole follow-up period. Furthermore, data from primary care were not available. But, although the data concerning health care use and prescription drug use is an underestimation of the true use of health care and prescription drugs, there is no reason to believe that the lack of data affected any of the groups in particular. Thus, the comparison between the groups should be valid.

Another possible limitation is that the original community study was designed to screen for depression and not for hypomania. However, 317 controls with negative screening were also diagnostically interviewed. In addition, numerous studies [27,57,82-87] have demonstrated that early onset of depressive disorders in children or adolescents usually precedes bipolar disorder. Still, there are a few studies [6,21] that have reported atypical mania-like symptoms in young children, mainly comorbid with ADHD. A fully representative sample of adolescents with hypomania spectrum episodes might show a slightly different comorbidity pattern in adulthood.

Further, there is a risk that we failed to identify important confounders. We adjusted the analyses for sex, and the information we had on socio-economic status and social stressors indicated that there were no significant differences between the hypomania spectrum group and the MDD group. However, residual confounding is possible.

A further limitation is the general risk of type II errors because of the relatively small number of participants. Thus, it is not possible to conclude that the outcome of adolescents with hypomania spectrum and MDD is equivalent. However, the data suggest that there are no fundamental differences between the groups.

## Conclusions

We conclude that the adult health outcomes do not differ fundamentally between adolescents with hypomania spectrum episodes and adolescents with MDD. Thus, adolescent hypomania spectrum episodes might not be a marker for worse health outcomes among adolescents with mood disorders. However, adolescents with mood disorders in general are at increased risk of subsequent health problems and could benefit from better treatment and follow-up. Clinicians should use a developmentally sensitive approach to early detection, prevention and treatment of mood disorders in children, adolescents, and young adults.

## Abbreviations

DSM-IV: American Psychiatric Association Diagnostic and statistical manual of mental disorders; DICA: Diagnostic Interview for Children and Adolescents in the revised form according to DSM-III-R for adolescents; BDI-C: Beck Depression Inventory-Child; CES-DC: Centre for Epidemiological Studies – Depression Scale for Children; MINI: Mini International Neuropsychiatric Interview Plus; DIP-Q: DSM-IV – ICD-10 Personality Questionnaire; MDD: Major depression disorder; OCD: Obsessive-compulsive disorder; ADHD: Attention-deficit/hyperactivity disorder; CD: Conduct disorder; ODD: Oppositional defiant disorder; PTSD: Posttraumatic stress disorder; ATC: Anatomical therapeutic chemical classification system; DDDs: Defined Daily Doses, measurement units of prescription drugs.

## Competing interests

The authors declare that they have no competing interests.

## Authors’ contributions

AP had the original idea for this study, had primary responsibility for the data analysis, and drafted and revised the manuscript. UJ had primary responsibility for the data collection in the follow-up phase of the study, supervised the design and execution of the study, and contributed to the conception, analyses and interpretation of the data and to the revision of the manuscript. A-LvK supervised the design and execution of the study, and contributed to the conception, analyses and interpretation of the data and to the revision of the manuscript. GO had primary responsibility for the patient screening and enrollment in the baseline study, and contributed to the conception of the study, the analyses and interpretation of the data and to the revision of the manuscript. HB contributed to the conception of the study, the analyses and interpretation of the data and to the revision of the manuscript. LvK contributed to the conception of the study, the analyses and interpretation of the data and to the revision of the manuscript. All authors have approved of the final version of the manuscript.

## Pre-publication history

The pre-publication history for this paper can be accessed here:

http://www.biomedcentral.com/1471-244X/14/9/prepub

## Supplementary Material

Additional file 1: Table S1 Attrition analyses: mental comorbidity in adolescents with hypomania spectrum, major depressive disorder (MDD) and in non-mood controls, split into those who did not participate in the follow-up (attrition) and those who did participate (followed). Click here for file
