# Supplementary material for: Hypomania spectrum disorder in adolescence: a 15-year follow-up of non-mood morbidity in adulthood
Source: BMC Psychiatry. 2014 Jan 15;14:9. doi: 10.1186/1471-244X-14-9 (PMC3898212; doi:10.1186/1471-244X-14-9)
Supplement: Additional file 1: Table S1 — Attrition analyses: mental comorbidity in adolescents with hypomania spectrum, major depressive disorder (MDD) and in non-mood controls, split into those who did not participate in the follow-up (attrition) and those who did participate (followed). [file 1471-244X-14-9-S1.docx]

**Supplement 1.** Attrition analyses: mental comorbidity in adolescents with hypomania spectrum, major depressive disorder (MDD) and in non-mood controls, split into those who did not participate in the follow-up (attrition) and those who did participate (followed).

| **Comorbid diagnoses**  **in adolescence**  **(<18 years)** | **All hypomania spectrum**  **n = 90**  **(%)** | | **MDD**  **n = 197**  **(%)** | | **Controls**  **n = 229**  **(%)** | | **p-value attrition vs. followed data in hypomania spectrum group** | **p-value attrition vs. followed data in MDD group** | **p-value**  **attrition vs. followed data in control group** |
| --- | --- | --- | --- | --- | --- | --- | --- | --- | --- |
|  | **attrition**  **n=26** | **followed n=64** | **attrition n=67** | **followed n=130** | **attrition n=81** | **followed n=148** |  |  |  |
| Gender (female) | 17 (65) | 46 (72) | 56 (84) | 111 (85) | 61 (75) | 120 (81) | n.s. | n.s. | n.s. |
| Separation Anxiety disorder | 8 (31) | 24 (38) | 31 (46) | 46 (35) | 12 (15) | 14 (10) | n.s. | n.s. | n.s. |
| Avoidant disorder | 5 (19) | 9 (14) | 4 (6) | 19 (15) | 1 (1) | 3 (2) | n.s. | n.s. | n.s. |
| Overanxious disorder | 8 (31) | 19 (30) | 24 (36) | 50 (39) | 3 (4) | 7 (5) | n.s. | n.s. | n.s. |
| Panic Disorder | 1 (4) | 11 (17) | 11 (16) | 15 (12) | 1 (1) | 1 (1) | n.s. | n.s. | n.s. |
| OCD | 12 (46) | 14 (22) | 18 (27) | 29 (22) | 4 (5) | 6 (4) | p<0.05 | n.s. | n.s. |
| PTSD | 1 (4) | 2 (3) | 7 (10) | 4 (3) | 1 (1) | - | n.s. | p<0.05 | n.s. |
| Psychotic symptoms | 3 (12) | 6 (9) | 1 (2) | 5 (4) | - | - | n.s. | n.s. | n.s. |
| Anorexia nervosa | - | - | 2 (3) | 8 (6) | 1 (1) | - | n.s. | n.s. | n.s. |
| Bulimia nervosa | - | 1 (2) | 1 (2) | 2 (2) | - | - | n.s. | n.s. | n.s. |
| ADHD | 4 (15) | 9 (14) | 6 (9) | 14 (11) | 1 (1) | 1 (1) | n.s. | n.s. | n.s. |
| CD | 11 (42) | 18 (28) | 16 (24) | 23 (18) | 4 (5) | 6 (4) | n.s. | n.s. | n.s. |
| ODD | 1 (4) | 7 (11) | 8 (12) | 12 (9) | 1 (1) | 1 (1) | n.s. | n.s. | n.s. |
| Drug/Sniffing/Alcohol Abuse | 2 (8) | 10 (16) | 5 (8) | 11 (9) | 1 (1) | 1 (1) | n.s. | n.s. | n.s. |
| **Any comorbidity** | 22 (84) | 48 (75) | 53 (79) | 104 (80) | 25 (31) | 33 (22) | n.s. | n.s. | n.s. |

* <0.05; ** <0.01; ***<0.001

MDD: Major depressive disorder; OCD: Obsessive-compulsive disorder; PTSD: Posttraumatic stress disorder; ADHD: Attention-deficit/hyperactivity disorder; CD: Conduct disorder; ODD: Oppositional defiant disorder.
